# Perceptual integration rapidly activates dorsal visual pathway to guide local processing in early visual areas

**DOI:** 10.1371/journal.pbio.2003646

**Published:** 2017-11-30

**Authors:** Ling Liu, Fan Wang, Ke Zhou, Nai Ding, Huan Luo

**Affiliations:** 1 School of Psychological and Cognitive Sciences, Peking University, Beijing, China; 2 Peking University-IDG/McGovern Institute for Brain Research, Peking University, Beijing, China; 3 Beijing Key Laboratory of Behavior and Mental Health, Peking University, Beijing, China; 4 State Key Laboratory of Brain and Cognitive Science, Institute of Biophysics, Chinese Academy of Sciences, Beijing, China; 5 College of Psychology and Sociology, Shenzhen University, Shenzhen, China; 6 Center for Language and Brain, Shenzhen Institute of Neuroscience, Shenzhen, China; 7 College of Biomedical Engineering and Instrument Sciences, Zhejiang University, Hangzhou, China; 8 Key Laboratory for Biomedical Engineering of Ministry of Education, Zhejiang University, Hangzhou, China; 9 State Key Laboratory of Industrial Control Technology, Zhejiang University, Hangzhou, China; National Institute of Mental Health, United States of America

## Abstract

Rapidly grouping local elements into an organized object (i.e., perceptual integration) is a fundamental yet challenging task, especially in noisy contexts. Previous studies demonstrate that ventral visual pathway, which is widely known to mediate object recognition, engages in the process by conveying object-level information processed in high-level areas to modulate low-level sensory areas. Meanwhile, recent evidence suggests that the dorsal visual pathway, which is not typically attributable to object recognition, is also involved in the process. However, the underlying whole-brain fine spatiotemporal neuronal dynamics remains unknown. Here we used magnetoencephalography (MEG) recordings in combination with a temporal response function (TRF) approach to dissociate the time-resolved neuronal response that specifically tracks the perceptual grouping course. We demonstrate that perceptual integration initiates robust and rapid responses along the dorsal visual pathway in a reversed hierarchical manner, faster than the ventral pathway. Specifically, the anterior intraparietal sulcus (IPS) responds first (i.e., within 100 ms), followed by activities backpropagating along the dorsal pathway to early visual areas (EVAs). The IPS activity causally modulates the EVA response, even when the global form information is task-irrelevant. The IPS-to-EVA response profile fails to appear when the global form could not be perceived. Our results support the crucial function of the dorsal visual pathway in perceptual integration, by quickly extracting a coarse global template (i.e., an initial object representation) within first 100 ms to guide subsequent local sensory processing so that the ambiguities in the visual inputs can be efficiently resolved.

## Author summary

How the brain integrates local elements into a global object (i.e., perceptual integration) in noisy contexts constitutes a fundamental yet challenging question in cognitive neuroscience. Here, we recorded brain activity by using magnetoencephalography from human subjects watching glass-pattern stimuli to examine the fine spatiotemporal neuronal responses during perceptual integration. We demonstrate that high-level brain regions initially extract a coarse global form of the inputs, which is then relayed along the dorsal visual pathway in a reversed hierarchical manner to low-level areas to modulate local analysis. This global-to-local modulation mechanism is especially beneficial in noisy environments by rapidly making an “initial guess” to guide detail analysis so that the ambiguities in inputs can be efficiently resolved.

## Introduction

The visual system effectively integrates fragmented visual inputs into organized visual objects; a process known as perceptual integration [1]. This process is particularly critical when the global form (i.e., formed foreground figure) is embedded in a noisy background and needs to be quickly and efficiently extracted (e.g. [2,3]). The ventral occipitotemporal pathway, which is well established to subserve object perception and analysis (the “what”), has been found to engage in the perceptual integration process centrally. For example, the lateral occipital (LO) complex (LOC) region, a high-level ventral area that is associated with object recognition [4], serves as a critical stage to start the grouping process and to convey the object information along the ventral pathway in a reversed way to modulate activities in low-level sensory areas [5–8].

Meanwhile, recent accumulating evidence challenges the traditional view and instead supports a substantial involvement of the dorsal occipitoparietal pathway (see review by [9,10]), which is typically considered to represent object locations, spatial relationship, and visually guided action (the “where” and “how”) [11,12]. For instance, dorsal visual areas such as the intraparietal sulcus (IPS) and V3a are significantly activated during perceptual grouping [13] and object representation [14–16], which are processes relatively independent of action planning or execution. Parietal lesions [17] or transcranial magnetic stimulation on parietal areas [13] have been shown to disrupt the behavioral performance in perceptual grouping. In a complementary fashion, patients with lesions to the ventral pathway retain sensitivities to object structure information [18], supporting the relatively independent role of the dorsal pathway in object representation. However, taken together, the fine spatiotemporal structure of neuronal responses at a whole-brain level during perceptual integration has not yet been well characterized. It remains unknown whether the dorsal visual pathway merely receives downstream signals from the ventral visual pathway and is consequently activated during perceptual integration, or whether the dorsal pathway plays a rather independent function and engages in the process with distinct dynamics.

We used magnetoencephalography (MEG), a whole-brain imaging technique with excellent temporal resolution and good spatial resolution, to assess the whole-brain spatiotemporal characteristics of perceptual integration in human subjects. We utilized glass patterns stimuli [19] to examine how noisy local inputs are integrated into global shapes. The glass pattern, formed from the superimposition of multiple random dot patterns, generates a percept of global structure (e.g., circular, radial, etc.) by pooling local elements, and thus, can be used to trace the integration process. Crucially, we employed a temporal response function (TRF) method [20–22] to separate the neural tracking of perceptual integration and luminance from the same MEG recordings.

Our results consistently demonstrate that perceptual integration significantly and quickly activates the dorsal visual pathway, and most importantly, the activations in the dorsal pathway are essentially faster than the ventral visual pathway. Specifically, the process initiates in the anterior IPS region within the first 100 ms, followed by a reversed activation course occurring along the dorsal hierarchical pathway to finally reach the EVA, and the IPS causally modulates the EVA responses. The IPS-to-EVA response profile for global form processing is preserved even under task-irrelevant conditions, excluding the possibility that the dorsal pathway activation is an artifact of attentional modulation. Finally, the IPS-to-EVA pathway is not activated when the global form of the glass pattern stimuli cannot be successfully tracked. Our findings demonstrate that perceptual integration is essentially mediated by a reversed hierarchical dorsal pathway that begins in the IPS, which rapidly extracts the global structure of the visual display. The global information is then transmitted back along the dorsal pathway to the EVA, where the local information processing is efficiently guided and anchored in a global context.

## Results

### TRF analyses: Dissociating luminance and global form neural tracking

**Fig 1:**
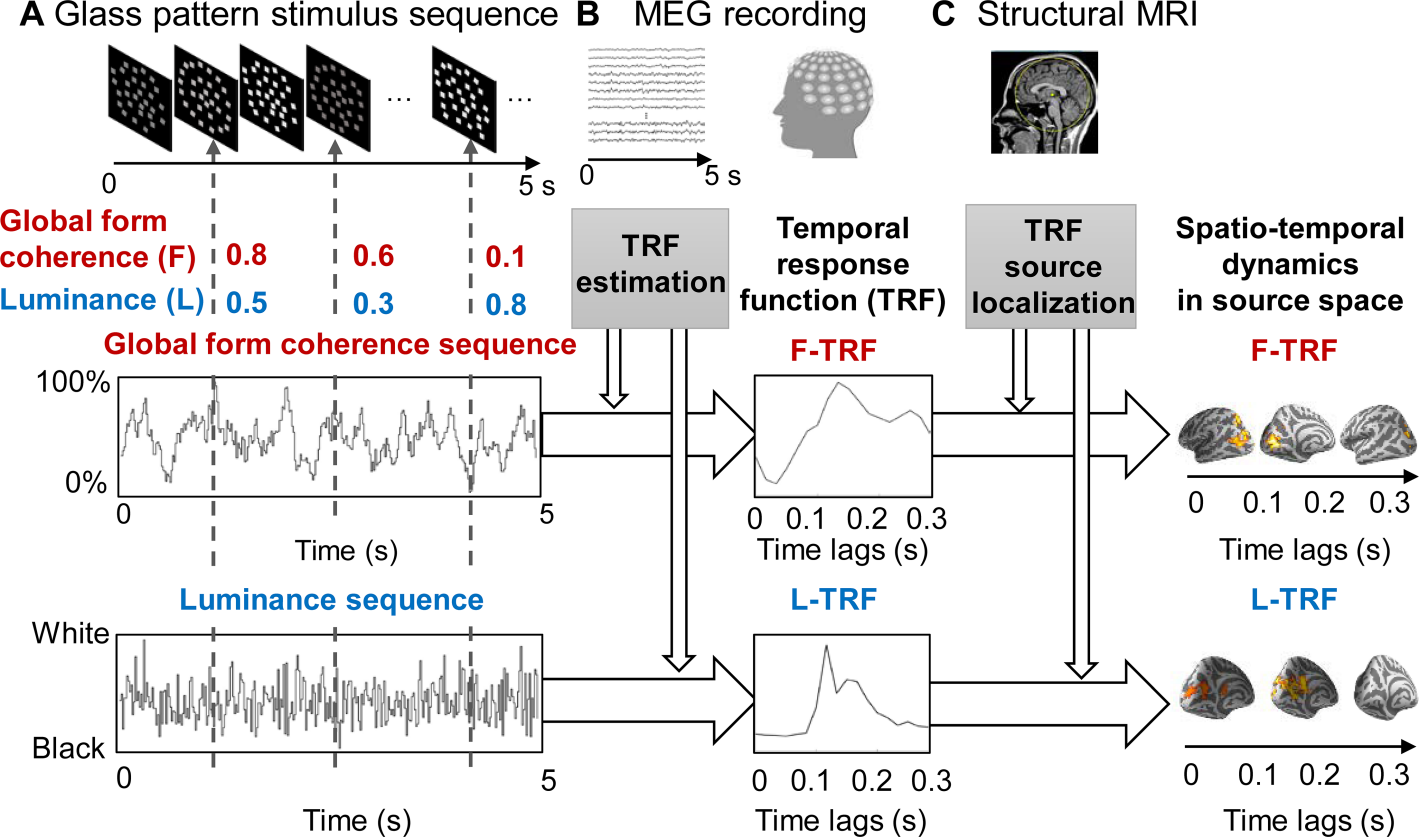
Experimental paradigm and illustration of the TRF approach. (A) We recorded 275-channel, whole-head magnetoencephalography signals from subjects who were viewing a 5-s circular glass pattern stimulus sequence in each trial. The global F (red) and the L (blue) of the glass pattern stimuli were continuously modulated according to 2 independent and randomly generated 5-s temporal sequences (the global F sequence and the L sequence) throughout each trial. (B) The F-TRF (red) and L-TRF (blue) responses were then calculated from the same MEG recordings based on the corresponding stimulus temporal sequences for each of the 275 MEG channels in each subject by using a linear least-squares approach. (C) Spatiotemporal dynamics of the F-TRF and L-TRF responses in source space (dSPM method for source localization) was calculated in combination with individual anatomical MRI scans. dSPM, dynamic statistical parametric mapping; F, form coherence; F-TRF, form coherence TRF; L, luminance; L-TRF, luminance TRF; MEG, magnetoencephalography; TRF, temporal response function.

We recorded 275-channel, whole-head MEG signals from 20 human subjects who viewed 5-s circular-shaped glass pattern stimulus sequences (Fig 1). We employed a TRF approach [20,21] to extract and isolate the neuronal responses that specifically tracks the ongoing changes in the global form coherence and luminance of the glass pattern stimuli throughout each trial. Specifically, 2 features—luminance and global form coherence—were modulated continuously by 2 independent 5-s sequences that were randomly generated in each trial (see Materials and methods and Fig 1, lower left panels). Next, the form coherence TRF (F-TRF) response and luminance TRF (L-TRF) response were calculated from the same MEG recordings for each of the 275 MEG channels in each subject (Fig 1, lower middle panel).

The TRF is defined as the brain response to a unit increment in the stimulus sequence. To assess the fine spatiotemporal patterns of the neuronal responses, we performed source analyses (dynamic statistical parametric mapping [dSPM] [23], function in Minimum Norm Current Estimates [MNE]-Python tools [24]) on the F-TRF and L-TRF, respectively, in combination with individual anatomical MRI (Fig 1, lower right panel).

### Experiment 1: Distinct neuronal spatiotemporal profiles for perceptual integration (F-TRF) and luminance (L-TRF) processing

**Fig 2:**
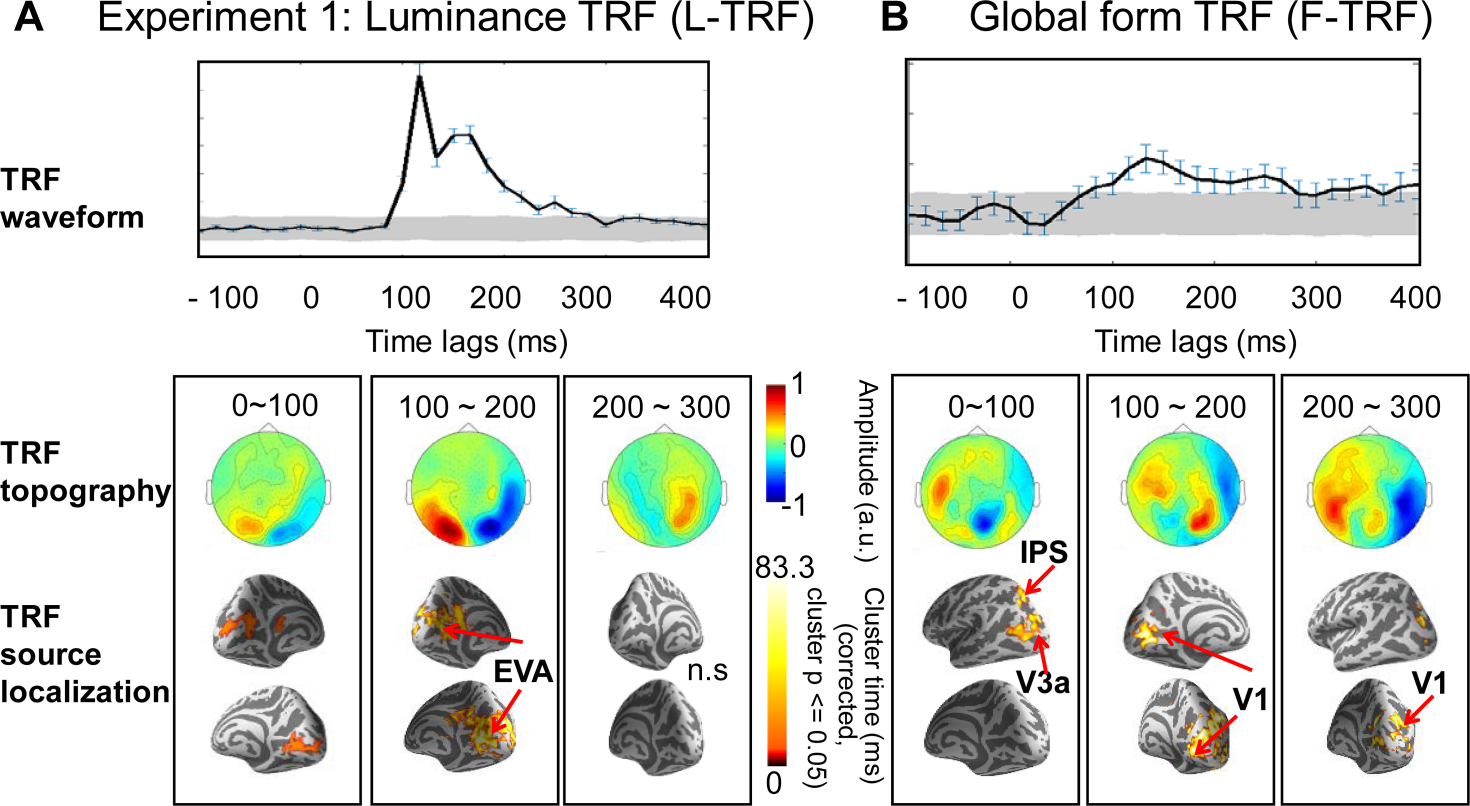
Experiment 1: L-TRF and F-TRF responses showed distinct neuronal spatiotemporal profiles. (A) L-TRF responses. (B) F-TRF responses. Note that the TRF responses represent brain responses to each unit transient in luminance (L-TRF) or global form coherence (F-TRF) of the glass pattern sequences. Upper panel: Grand average (*n* = 20) plots for TRF waveforms (summarized as root-mean-square across all MEG channels) as a function of temporal lag (−100 to 400 ms). Gray shades indicate the confidence interval after permutation test (see details in S1 Text). Error bar indicates the standard error. Middle panel: Grand average (*n* = 20) plots for sensor-level topographical distribution of TRF responses at 0–100, 100–200, and 200–300 ms time range. Lower panel: Grand average (*n* = 20) plots for TRF source localization results in the normalized MNI template at 0–100, 100–200, and 200–300 ms time range (cluster-level permutation test across space and time, multiple comparison corrected, cluster *p* < 0.05). The MNI coordinates for the significant source clusters are listed in S1 Table. It is notable that F-TRF activated the IPS and V3a within the first 100 ms, followed by responses in primary visual cortices in the next 100 ms, in accordance with a reversed high-to-low activation pattern. Meanwhile, L-TRF showed a feedforward profile that started from EVAs. The data underlying Fig 2 can be found in S1 Data. EVAs, early visual areas; F-TRF, form coherence TRF; IPS, intraparietal sulcus; L-TRF, luminance TRF; MEG, magnetoencephalography; MNI, Montreal Neurological Institute; n.s., not significant.

As illustrated in Fig 2, the F-TRF and L-TRF responses displayed distinct profiles in their temporal waveforms (upper panels), field topography maps (middle panels), and source-level spatiotemporal patterns (lower panels). Specifically, luminance processing (L-TRF) initially started in the EVAs around 100 ms after each luminance change (Fig 2A, cluster-level permutation *t* test (versus baseline) across space and time, multiple comparisons corrected, cluster *p* < 0.05), suggesting a classical feedforward course. In contrast, global form processing (F-TRF) displayed a rather reversed high-to-low activation pattern (Fig 2B). Specifically, global form processing appeared to first activate high-level brain regions, such as the anterior IPS (Montreal Neurological Institute [MNI] coordinates: −18, −65, 50) and V3a, within the initial 100 ms (cluster *p* < 0.05, corrected), followed by subsequent responses in the low-level EVAs in the subsequent 200 ms (cluster *p* < 0.05, corrected). The anatomical coordinates of the source localizations are listed in S1 Table. Moreover, to further examine the ventral pathway response, although no regions of interest (ROIs) in the ventral visual pathway were significant in the whole-brain analysis, using small volume correction (SVC) analysis [25], we localized a cluster in the LO area for F-TRF responses.

**Fig 3:**
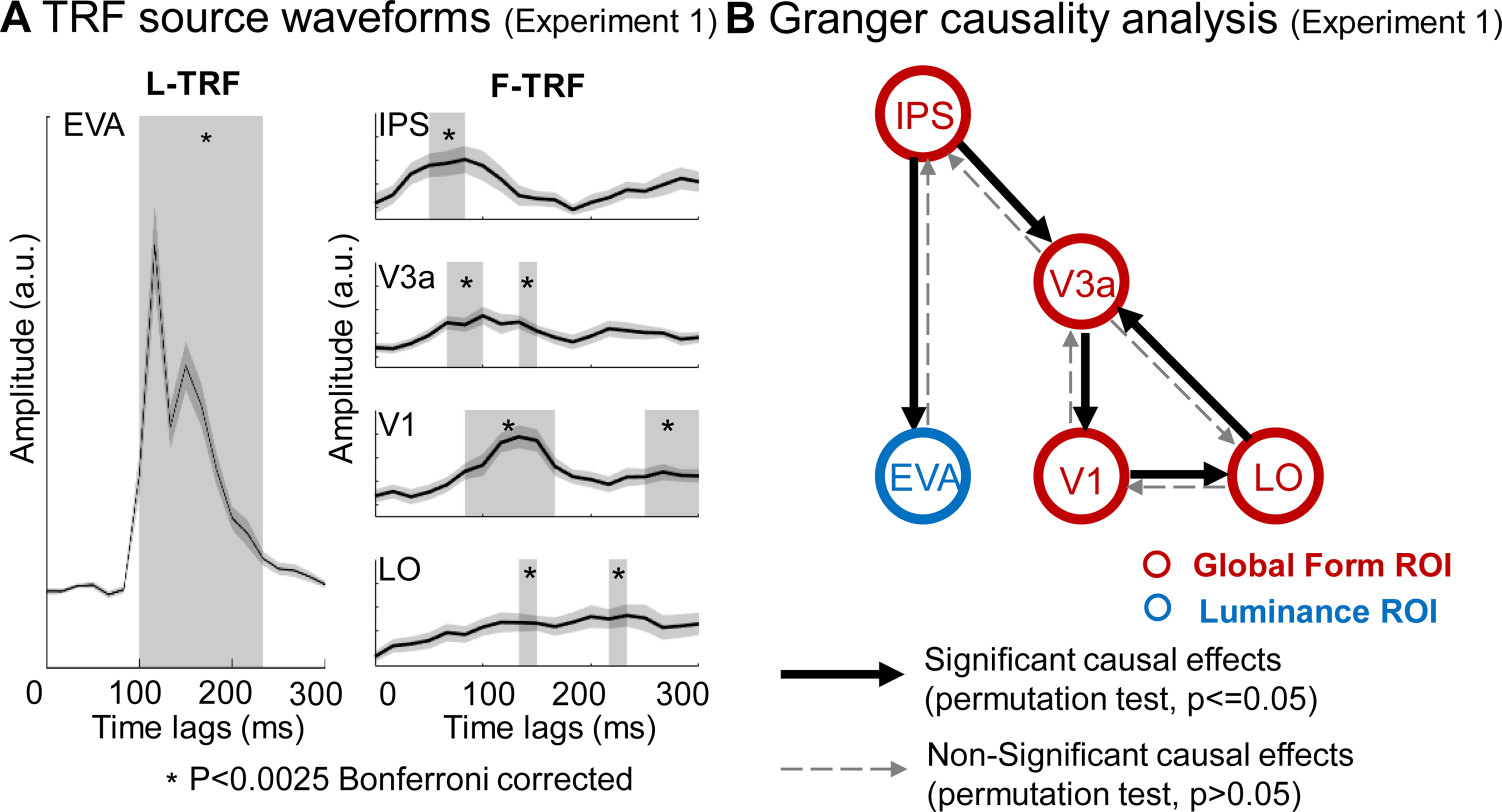
Experiment 1: TRF ROI source waveforms and granger causality analysis. (A) Grand average (*n* = 20) plots for TRF source waveforms in the ROIs, defined according to source localization results, for L-TRF (left column) and F-TRF (right column) responses. Gray box indicates time ranges when the TRF responses showed significant activations compared to baseline (*p* < 0.0025, Bonferroni corrected). It is notable that for F-TRF responses (right column), IPS, V3a, V1 showed sequential activations (right panel), supporting a reversed hierarchical activation profile. (B) Granger causality analysis of activation time courses among ROIs for F-TRF (red) and L-TRF (blue). The solid arrows indicate significant causal effects (*p* < 0.05, permutation test), whereas the dashed arrows indicate nonsignificant causal effects. The data underlying Fig 3 can be found in S1 Data. EVA, early visual area; F-TRF, form coherence TRF; IPS, intraparietal sulcus; LO, lateral occipital; L-TRF, luminance TRF; ROIs, regions of interest; TRF, temporal response function.

Next, to assess the fine spatiotemporal patterns of the neuronal responses, we conducted another analysis in source space to extract the fine activation time courses of the F-TRF and L-TRF in each ROI. For global form processing (Fig 3A, right panel), the source activity in the IPS began to increase around 50 ms (*t* test versus baseline, *p* < 0.0025, Bonferroni corrected), and it then activated V3a 20 ms later (around 70 ms, *p* < 0.0025, corrected) and V1 35 ms later (around 85 ms, *p* < 0.0025, corrected), suggesting a reversed IPS-driven activation profile for global form processing. Notably, the LO responses started around 130 ms (*p* < 0.0025, corrected), later than IPS and V1 (Fig 3). In contrast, the source activity for luminance processing was first initiated in the EVAs around 100 ms (*p* < 0.0025, corrected), consistent with a feedforward response course (Fig 3A, left panel). Taken together, the source-level analysis results confirm that luminance and global form processing are associated with distinct neuronal pathways: a low-to-high feedforward pathway and high-to-low feedback pathway, respectively.

We next performed a Granger causality analysis [26] on the source waveforms. During global form processing, the IPS causally exerted influences on V3a, which subsequently resulted in EVA activation that further modulated LO area, supporting the IPS-initiating modulation of low-level sensory cortical responses (Fig 3B). We also examined the causal relationship between F-TRF and L-TRF responses in source space, and observed significant form-to-luminance driving effects (Fig 3B). Specifically, the IPS activations during global form processing (red circle) significantly affected the luminance processing (blue circle) in EVA. In summary, the IPS initiates global form processing and guides luminance processing in the EVAs.

### Experiment 2: Perceptual integration processing under task-irrelevant conditions

The results described above suggest that global form processing is associated with a high-to-low feedback pathway that starts in IPS. However, IPS is also known to play an important role in selective attention [27,28], whereas in Experiment 1, subjects were instructed to detect change in global form shape. Thus, the specific IPS activation during global form processing might have been due to top-down task-relevant attentional enhancement (i.e., attending to the global form).

**Fig 4:**
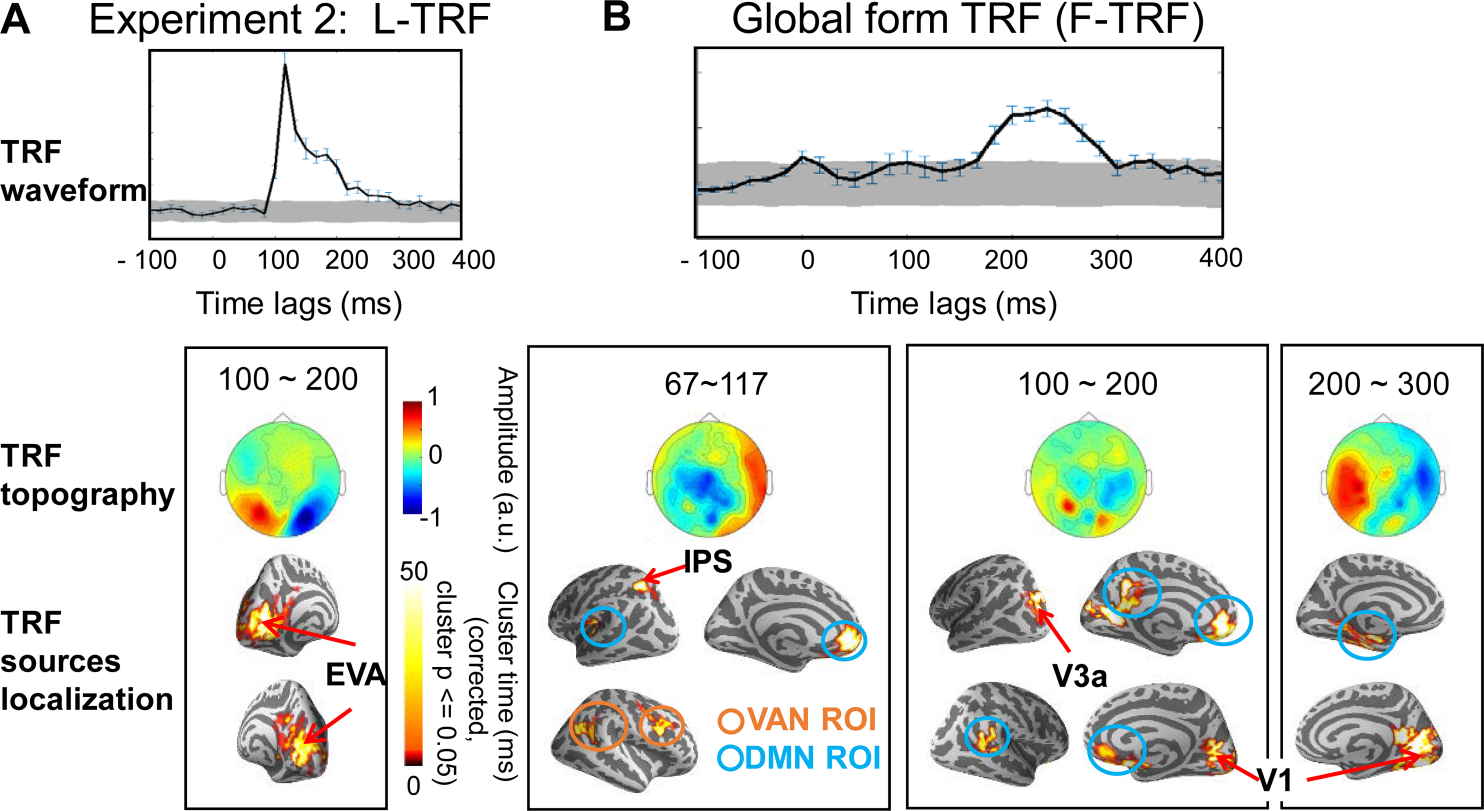
Experiment 2: L-TRF and F-TRF responses when global form property was task irrelevant. (A) L-TRF responses. (B) F-TRF responses. Upper panel: Grand average (*n* = 16) plots for TRF waveforms (summarized as root-mean-square across all MEG channels) as a function of temporal lag (−100 to 400 ms). Gray shades indicate the confidence interval after permutation test (see details in S1 Text). Error bar indicates standard error. Middle panel: Grand average (*n* = 16) plots for sensor-level topographical distribution for TRF responses. Lower panel: Grand average (*n* = 16) plots for source localization results in the normalized MNI template (cluster-level permutation test across space and time, multiple comparison corrected, cluster *p* < 0.05). Note that L-TRF responses showed a similar feedforward profile that started from EVA as that in Experiment 1 (A). Crucially, the IPS-V3a-V1 activation sequence still emerged although was temporally delayed (B). Moreover, the VAN (orange) and DMN (blue) were also activated. The MNI coordinates for all the significant source clusters are listed in S1 Table. The data underlying Fig 4 can be found in S1 Data. DMN, default mode network; F-TRF, form coherence TRF; IPS, intraparietal sulcus; L-TRF, luminance TRF; MEG, magnetoencephalography; MNI, Montreal Neurological Institute; ROI, region of interest; TRF, temporal response function; VAN, ventral attention network.

**Fig 5:**
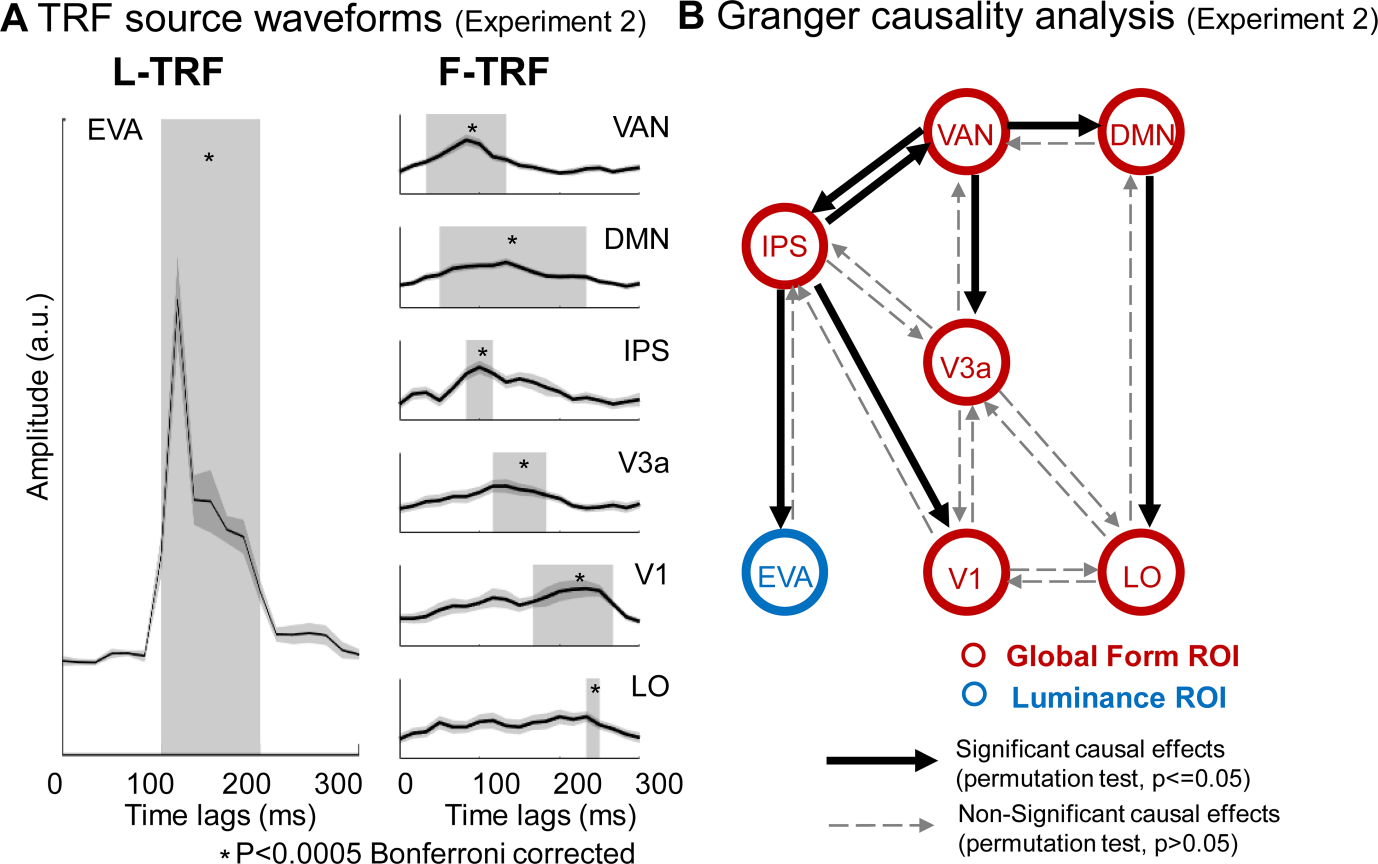
Experiment 2: TRF ROI source waveforms and granger causality analysis. (A) Grand average (*n* = 16) plots for TRF source waveforms in the ROIs, defined according to source localization results, for L-TRF (left column) and F-TRF (right column) responses. Gray box indicates time ranges when the TRF responses showed significant activations compared to baseline (*p* < 0.0005, Bonferroni corrected). It is notable that for F-TRF responses (right column), IPS, V3a, V1, and LO showed sequential activations (right panel), which is consistent with the reversed hierarchical activation profile found in Experiment 1. Moreover, VAN and DMN showed earlier responses than IPS. (B) Granger causality analysis of activation time courses among ROIs for F-TRF (red) and L-TRF (blue). The solid arrows indicate significant causal effects (*p* < 0.05, permutation test); the dashed arrows indicate non-significant causal effects. The data underlying Fig 5 can be found in S1 Data. DMN, default mode network; EVA, early visual area; F-TRF, form coherence TRF; IPS, intraparietal sulcus; LO, lateral occipital; L-TRF, luminance TRF; IPS, intraparietal sulcus; ROIs, regions of interest; TRF, temporal response function; VAN, ventral attention network.

To further examine the issue, 16 subjects were recruited in Experiment 2 and were presented with the same glass pattern stimuli as in Experiment 1. However, they were required to perform a global form irrelevant task by monitoring an abrupt full-screen luminance change. As shown in Figs 4 and 5, global form processing (F-TRF) elicited similar reversed high-to-low activation profiles that started in the anterior IPS (MNI coordinates: −25, −59, 48). The IPS activation was then followed by sequential responses in V3a, V1, and LO, similar to Experiment 1. Thus, task-relevant attentional modulation (i.e., attending to the global form) alone could not account for the observed IPS activation during global form processing. Instead, the IPS appears to be critical for initiating perceptual integration and subsequently guiding the processing in the EVAs, independent of task-modulated attention.

Furthermore, areas in ventral attention network (VAN) and default mode network (DMN) [27–29], which are known to be involved in inhibiting irrelevant features during attentional control [28,30,31], were also activated during global form processing here (see details in S1 Table). Interestingly, the IPS-to-EVA response profile was temporally delayed, compared to Experiment 1. Specifically, activation of the IPS, V3a, and V1 was initiated around 80 ms, 100 ms, and 150 ms, respectively (*p* < 0.0005, corrected), which was approximately 30 ms later than the activation found in Experiment 1. Taken together, these results suggest that, when global form feature is task irrelevant and does not need to be attended to, the VAN and DMN is quickly activated to inhibit the task-irrelevant global form neuronal pathway, which in turn has a temporally delayed response.

Granger causality analysis on the source waveforms demonstrate that for global form processing (Fig 5B, red), the VAN drove activation in the IPS, V3a, and DMN, and the DMN further modulated the LO, reflecting the task-dependent inhibition of the global form processing. Importantly, consistent with Experiment 1, the IPS causally modulated the V1, and the global form processing in the IPS significantly guided the luminance processing in the EVAs (Fig 5B, blue).

### Experiment 3 and rate control

**Fig 6:**
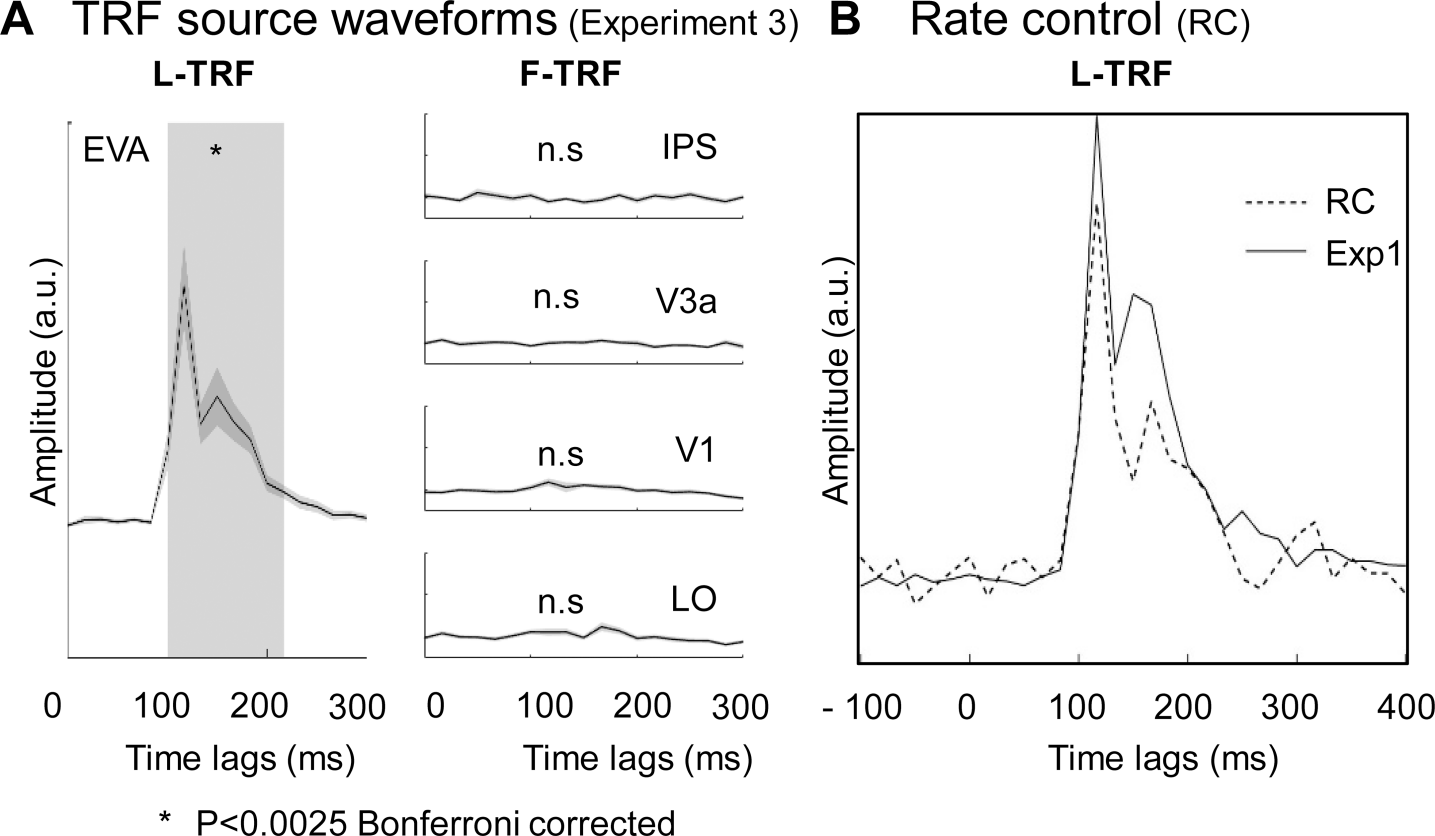
Experiment 3 and rate control experiment. (A) Experiment 3: untracked global form did not activate the IPS-to-V1 neuronal pathway. Grand average (*n* = 16) plots for TRF source waveforms in the ROIs (IPS, V3a, V1, and LO) for L-TRF (left column) and F-TRF (right column) responses. The gray area indicates time points when the TRF showed significant activations compared to baseline activities (*p* < 0.0025, Bonferroni corrected). Note the nonsignificant activations in IPS-to-V3a-to-V1 pathway for F-TRF responses (right column). (B) Results for rate control experiment. Grand averaged plots for L-TRF response under fast luminance modulation condition (solid line, Experiment 1 data, *n* = 20) and slow luminance modulation condition (dashed line, rate control, *n* = 4). The 2 L-TRF responses showed no difference, suggesting that the obtained TRF response is independent of modulation rate of the temporal sequence of the stimuli. The data underlying Fig 6 can be found in S1 Data. EVA, early visual area; Exp1, Experiment 1; F-TRF, form coherence TRF; IPS, intraparietal sulcus; LO, lateral occipital; L-TRF, luminance TRF; n.s., not significant; TRF, temporal response function.

Notably, in Experiment 1 and 2, the modulation speed of the global form coherence was slowed down by smoothing (8 points) the stimulus sequences to ensure that the subjects were able to clearly track the modulations in global form coherence (see Fig 1). In Experiment 3, we employed glass pattern stimuli with global form coherence sequences that were not temporally smoothed as before, and in consequence, subjects reported that they were not able to track or identify the ongoing global form fluctuations. As illustrated in Fig 6A, the new stimuli did not activate the IPS and V3a, supporting a central role of the IPS-to-EVA neuronal profile in global form processing. These results imply that the less robustly the subjects track the global form coherence, the less activated the neuronal pathway is.

Furthermore, we also slowed down the modulation rate of luminance by temporally smoothing (8 points) the luminance temporal sequence. As shown in Fig 6B, the spatiotemporal profiles of the new L-TRF responses were similar to those observed in the previous experiments, suggesting that the computed TRF responses were independent of the temporal modulation rates of the stimulus sequences.

## Discussion

We used MEG recordings in human subjects to track the fine spatiotemporal characteristics of the perceptual integration process using visual glass pattern stimuli. A TRF technique was employed to extract the neural responses that specifically detect the ongoing changes in the coherence of the global form of the visual stimuli to delineate the neural signature for perceptual integration. We demonstrate a fast activation courses in dorsal visual pathway during the grouping process, compared to the ventral pathway. Specifically, it starts with rapid activation (within the first 100 ms) in the anterior IPS, and the responses then quickly propagate through a reverse hierarchy to the EVAs by successively modulating activation of the lower stages along the dorsal visual pathway. The progressive IPS-to-EVA activations occur in both task-relevant and task-irrelevant conditions, but do not appear when the global form could not be successfully perceived or tracked. Our results support an independent and crucial role of the dorsal visual pathway in perceptual integration. Taken together, in a noisy circumstance, the brain rapidly extracts a coarse global form (i.e., dot coherence) in high-level dorsal visual regions (i.e., the IPS), potentially through rapid magnocellular signals or the subcortical pathway. The IPS responses then convey the coarse “initial guess” about the object’s structure to guide subsequent local sensory processing (i.e., the EVAs), so that local ambiguities and conflicting information that is embedded in the cluttered visual inputs can be efficiently resolved.

One key result is the central involvement of dorsal visual pathway, especially the IPS region, in perceptual integration. Recent studies and theoretical frameworks advocate a crucial and irreplaceable role of parietal cortices in top-down modulation during visual information processing [32–35]. For example, parietal cortices process information about global-level category [36], low spatial frequency [37], attentional modulation [28], visual object unit [14], perceptual rivalry [38], priority map [39], visual ensembles (summary statistics) [40], and task information [41]. Furthermore, global form processing is disrupted by lesions in the IPS but not in V1 [17], and transcranial magnetic stimulation of the IPS also interfere with perceptual integration performance [13]. Our results are thus consistent with these findings but also provide the novel neural evidence advocating that the IPS area and the dorsal visual pathway principally initiates and drives the perceptual integration process, rather than merely acting as a downstream recipient of ventral activations.

Using MEG recordings that could monitor the time-dependent details of the brain activities, we found that the IPS is rapidly activated within the first 100 ms during global form processing, earlier than other cortical areas. Previous neurophysiological recordings have revealed that information quickly reaches the lateral intraparietal cortex within 50 ms to guide spatial attention [42] and saccades [43], constituting possible neurophysiological evidence. In fact, 2 possible fast neural pathways might account for and mediate the rapid IPS activations: a subcortical pathway to the IPS through the superior colliculus (SC) and pulvinar [44] and a magnocellular cortical pathway that links V1 to the lateral intraparietal cortex through the medial temporal area [45]. This rapid IPS activation is also consistent with previous electroencephalography (EEG) recordings for glass pattern stimuli [46], which revealed that the poster electrode activity at 90 ms poststimulus marks the awareness of global structure.

We also dissociated the involvement of the IPS in perceptual integration from selective attention [27,28]. The reversed IPS-to-EVA activation profile was preserved even when the global form was task irrelevant. Meanwhile, task-modulated attention still influenced the integration process, and the IPS-to-EVA activation time course was temporally delayed by approximately 40 ms. These delayed responses were mainly due to inhibition by the VAN and DMN, which are neural networks that are affiliated with task-irrelevant feature inhibition, consistent with a recent study demonstrating that responses for task-irrelevant features were temporally delayed [47]. It is notable that in Experiment 2, the global form property is task irrelevant throughout the whole experiment (i.e., a block design), and VAN and DMN may therefore be activated to inhibit the global form property in a sustained way. Furthermore, IPS was not activated when perceptual integration could not be successfully achieved, which is also in line with previous work showing that perceptual grouping did not occur under inattentional blindness [48]. Thus, the brain automatically computes the global structures, regardless of the task contexts or demands and the IPS-to-EVA pathway is modulated by classical attention networks (e.g., delayed response under task-irrelevant conditions). Moreover, we observed a left lateralization for IPS activations, also consistent with previous finding disclosing significant associations between left IPS and saliency coding [49–51].

We postulate that IPS activation here could not be accounted for by eye movement profiles. First, if the 3 experiments were associated with different eye movement profile, we would expect to find activations in frontal eye field region, which we did not observe here in all the 3 experiments. Moreover, evoked eye movements are known to elicit brain activity around 130–170 ms after stimulus onset [52,53], which could not interpret the early IPS activations. Further eye movement recordings showed that participants were able to maintain central fixation (i.e., deviation in eye position did not exceed 0.70 degrees) and the eye movement profiles did not significantly differ (F = .968, *p* = 0.416) for the 3 task conditions.

Why is the coarse global form, rather than the local detail, computed first? Notably, local information in a noisy visual environment typically evokes multiple possible interpretations, and thus to efficiently process complex visual scenes, the brain needs to generate a rapid initial guess [54], perceptual prediction [32], and coarse organization of the discrete objects [14] for the noisy visual inputs. Actually, the visual objects, rather than the simple local features, are thought to be the basic unit for visual perception and attention [55–58].

Finally, our results showed early activations in the dorsal pathway, consistent with a previous MEG study [59], but are different from studies revealing activations mainly in the ventral pathway (e.g., LOC, V4, etc.) [6–8,60]. Indeed, we found later activation of the ventral visual pathway compared to the dorsal visual pathway (Fig 3 and Fig 5). This discrepancy might have 2 explanations. First, we rapidly modulated the global form coherence of the pattern stimuli throughout the trial, and this type of dynamic stimulus would presumably drive the dorsal pathway more efficiently than the ventral pathway [61]. Second, the global form coherence corresponds to the strength of the perceptual integration rather than the outcome of the perceptual integration (i.e., perceived global form or shape). The results further support the idea that dorsal pathway provides an early coarse representation (global form) to the ventral pathway to guide and facilitate detailed shape and object recognition [9,62].

## Materials and methods

A detailed description of the methods is provided in S1 Text, Supplementary materials and methods, as part of supporting information.

### Ethics statement

Written informed consent was provided by all participants. Experiments protocols were approved by the Research Ethics Committee of Peking University (2015-03-05c2) and adhered to the principles conveyed in the Declaration of Helsinki.

### Participants

Twenty, 16, 16, and 4 subjects participated in Experiment 1, Experiment 2, Experiment 3, and the rate control experiment, respectively. All subjects had normal or corrected-to-normal vision and provided informed consent. The minimum sample size for Experiment 2 and Experiment 3 was decided based on the IPS effects in Experiment 1.

### Stimuli and tasks

To extract the impulse brain response using TRF technique, the luminance and the global form coherence (i.e., reflecting the saliency of perceptual integration) of the glass pattern stimuli were respectively modulated in time, according to the corresponding temporal sequence that was randomly and independently generated for each trial. In Experiment 1, subjects were instructed to monitor a brief circular-to-radial global form change. In Experiment 2, subjects were instructed to monitor a brief overall large luminance change. In Experiment 3, subjects performed the same task as in Experiment 1 by detecting a circular-to-radial global form change (see S1 Movie).

### MEG data acquisition and analysis

Neuromagnetic signals were recorded continuously with a 275-channel, whole-heard MEG system (axial gradiometer SQUID-based sensory; CTF MEG International Services LP, Coquitlam, British Columbia, Canada) in a magnetically shielded room. Each subject underwent anatomical MRI scans on a 3T Siemens Prisma scanner after MEG recordings. The TRF responses for global form coherence (F-TRF) and luminance (L-TRF) were calculated from the same MEG recordings, and were baseline corrected (see Fig 2 and S1 Fig). Next, source modeling was performed on the sensor-space TRF responses to examine the source localization. A cluster-level permutation test across space and time was performed to correct multiple comparisons, resulting in statistically significant clusters in source space. Finally, the source-level neuronal activation profile for each subject was normalized to MNI coordinates. Based on the source localization results in the whole brain analysis in combination with the anatomical landmarks in SVC analysis, ROIs for the F-TRF and L-TRF responses were separately defined and the corresponding activation time coursed were extracted. Granger causality analysis was performed on the extracted ROI-based activation time courses using the Multivariate Granger Causality toolbox [26]. The theoretical and null distributions for all pairs were compared with Kolmogorov-Smirnov tests.

## Supporting information

S1 Text Supplementary materials and methods. (DOCX)

S1 Fig ERF responses and the temporal range of responses for TRF calculation. Top: ERFs for all MEG channels as a function of time (0–5 s) after trial onset. Bottom: Source localization of the initial ERF onset responses (0–0.5 s). To avoid possible influence from the onset and offset responses, which may bias the estimated TRF results, we extracted the middle part (red rectangle) of the 5-s MEG trial responses (0.5–4.5 s) for further TRF calculation. Notably, the data segments for further TRF calculation showed a rather noisy and flat response pattern. ERF, event-related magnetic field; MEG, magnetoencephalography; TRF, temporal response function.
(TIF)

S1 Table MNI coordinates of the ROIs in the experiments. AG, angular gyrus; DMN, default mode network; EVAs, early visual areas; F-TRF, global form TRF; IPS, intraparietal sulcus; L-TRF, luminance TRF; LO, lateral occipital; MFG, middle frontal gyrus; MNI, Montreal Neurological Institute; mPFC, medial prefrontal cortex; PCC, posterior cingulate cortex; ROIs, regions of interest; TPJ, temporoparietal junction; VAN, ventral attention network.
(XLSX)

S1 Movie Example movie of the stimulus in Experiment 1. (GIF)

S1 Data Data for Figs 2, 3, 4, 5, 6A and 6B. (XLSX)

## Abbreviations

DMN: default mode network
dSPM: dynamic statistical parametric mapping
EEG: electroencephalography
EVA: early visual area
F-TRF: form coherence TRF
IPS: intraparietal sulcus
LO: lateral occipital
LOC: lateral occipital complex
L-TRF: luminance TRF
MEG: magnetoencephalography
MNE: Minimum Norm Current Estimates
MNI: Montreal Neurological Institute
ROI: region of interest
SC: superior colliculus
SVC: small volume correction
TRF: temporal response function
VAN: ventral attention network

## References

[1] Wertheimer M. Untersuchungen zur Lehre von der Gestalt. II. Psychologische Forschung. 1923;4(1):301–50. doi: 10.1007/bf00410640

[2] Thorpe S, Fize D, Marlot C. Speed of processing in the human visual system. Nature. 1996;381(6582):520–2. doi: 10.1038/381520a0 . 8632824 10.1038/381520a0

[3] Fabre-Thorpe M, Delorme A, Marlot C, Thorpe S. A limit to the speed of processing in ultra-rapid visual categorization of novel natural scenes. J Cogn Neurosci. 2001;13(2):171–80. . 11244543 10.1162/089892901564234

[4] Grill-Spector K, Kourtzi Z, Kanwisher N. The lateral occipital complex and its role in object recognition. Vision Res. 2001;41(10–11):1409–22. . 11322983 10.1016/s0042-6989(01)00073-6

[5] Murray SO, Schrater P, Kersten D. Perceptual grouping and the interactions between visual cortical areas. Neural Netw. 2004;17(5–6):695–705. doi: 10.1016/j.neunet.2004.03.010 . 15288893 10.1016/j.neunet.2004.03.010

[6] Shpaner M, Molholm S, Forde E, Foxe JJ. Disambiguating the roles of area V1 and the lateral occipital complex (LOC) in contour integration. Neuroimage. 2013;69:146–56. doi: 10.1016/j.neuroimage.2012.11.023 ; PubMed Central PMCID: PMCPMC3872825. 23201366 10.1016/j.neuroimage.2012.11.023 PMC3872825

[7] Mijovic B, De Vos M, Vanderperren K, Machilsen B, Sunaert S, Van Huffel S, et al The dynamics of contour integration: A simultaneous EEG-fMRI study. Neuroimage. 2014;88:10–21. doi: 10.1016/j.neuroimage.2013.11.032 . 24269572 10.1016/j.neuroimage.2013.11.032

[8] Fang F, Kersten D, Murray SO. Perceptual grouping and inverse fMRI activity patterns in human visual cortex. J Vis. 2008;8(7):2 1–9. doi: 10.1167/8.7.2 . 19146235 10.1167/8.7.2

[9] Freud E, Plaut DC, Behrmann M. 'What' Is Happening in the Dorsal Visual Pathway. Trends Cogn Sci. 2016;20(10):773–84. doi: 10.1016/j.tics.2016.08.003 . 27615805 10.1016/j.tics.2016.08.003

[10] Kastner S, Chen Q, Jeong SK, Mruczek RE. A brief comparative review of primate posterior parietal cortex: A novel hypothesis on the human toolmaker. Neuropsychologia. 2017 doi: 10.1016/j.neuropsychologia.2017.01.034 . 28159617 10.1016/j.neuropsychologia.2017.01.034 PMC5537042

[11] Ungerleider LGaM M. Two cortical visual systems. Analysis of visual behavior. 1982:549–86.

[12] Goodale MA, Milner AD. Separate visual pathways for perception and action. Trends Neurosci. 1992;15(1):20–5. . 1374953 10.1016/0166-2236(92)90344-8

[13] Zaretskaya N, Anstis S, Bartels A. Parietal cortex mediates conscious perception of illusory gestalt. J Neurosci. 2013;33(2):523–31. doi: 10.1523/JNEUROSCI.2905-12.2013 . 23303932 10.1523/JNEUROSCI.2905-12.2013 PMC6704896

[14] Xu Y, Chun MM. Visual grouping in human parietal cortex. Proc Natl Acad Sci U S A. 2007;104(47):18766–71. doi: 10.1073/pnas.0705618104 ; PubMed Central PMCID: PMCPMC2141851. 17998539 10.1073/pnas.0705618104 PMC2141851

[15] Freud E, Ganel T, Shelef I, Hammer MD, Avidan G, Behrmann M. Three-Dimensional Representations of Objects in Dorsal Cortex are Dissociable from Those in Ventral Cortex. Cereb Cortex. 2017;27(1):422–34. doi: 10.1093/cercor/bhv229 . 26483400 10.1093/cercor/bhv229 PMC13100970

[16] Konen CS, Kastner S. Two hierarchically organized neural systems for object information in human visual cortex. Nat Neurosci. 2008;11(2):224–31. doi: 10.1038/nn2036 . 18193041 10.1038/nn2036

[17] Lestou V, Lam JM, Humphreys K, Kourtzi Z, Humphreys GW. A dorsal visual route necessary for global form perception: evidence from neuropsychological fMRI. J Cogn Neurosci. 2014;26(3):621–34. doi: 10.1162/jocn_a_00489 . 24047377 10.1162/jocn_a_00489

[18] Behrmann M, Kimchi R. What does visual agnosia tell us about perceptual organization and its relationship to object perception? J Exp Psychol Hum Percept Perform. 2003;29(1):19–42. . 12669745 10.1037//0096-1523.29.1.19

[19] Glass L. Moire effect from random dots. Nature. 1969;223(5206):578–80. . 5799528 10.1038/223578a0

[20] Ding N, Simon JZ. Emergence of neural encoding of auditory objects while listening to competing speakers. Proc Natl Acad Sci U S A. 2012;109(29):11854–9. doi: 10.1073/pnas.1205381109 ; PubMed Central PMCID: PMCPMC3406818. 22753470 10.1073/pnas.1205381109 PMC3406818

[21] Di Liberto GM, O'Sullivan JA, Lalor EC. Low-Frequency Cortical Entrainment to Speech Reflects Phoneme-Level Processing. Curr Biol. 2015;25(19):2457–65. doi: 10.1016/j.cub.2015.08.030 . 26412129 10.1016/j.cub.2015.08.030

[22] Jia J, Liu L, Fang F, Luo H. Sequential sampling of visual objects during sustained attention. PLoS Biol. 2017;15(6):e2001903 doi: 10.1371/journal.pbio.2001903 . 28658261 10.1371/journal.pbio.2001903 PMC5489144

[23] Dale AM, Liu AK, Fischl BR, Buckner RL, Belliveau JW, Lewine JD, et al Dynamic statistical parametric mapping: combining fMRI and MEG for high-resolution imaging of cortical activity. Neuron. 2000;26(1):55–67. . 10798392 10.1016/s0896-6273(00)81138-1

[24] Gramfort A, Luessi M, Larson E, Engemann DA, Strohmeier D, Brodbeck C, et al MEG and EEG data analysis with MNE-Python. Front Neurosci. 2013;7:267 doi: 10.3389/fnins.2013.00267 ; PubMed Central PMCID: PMCPMC3872725. 24431986 10.3389/fnins.2013.00267 PMC3872725

[25] Van Essen DC. A Population-Average, Landmark- and Surface-based (PALS) atlas of human cerebral cortex. Neuroimage. 2005;28(3):635–62. doi: 10.1016/j.neuroimage.2005.06.058 . 16172003 10.1016/j.neuroimage.2005.06.058

[26] Barnett L, Seth AK. The MVGC multivariate Granger causality toolbox: a new approach to Granger-causal inference. J Neurosci Methods. 2014;223:50–68. doi: 10.1016/j.jneumeth.2013.10.018 . 24200508 10.1016/j.jneumeth.2013.10.018

[27] Corbetta M, Shulman GL. Control of goal-directed and stimulus-driven attention in the brain. Nat Rev Neurosci. 2002;3(3):201–15. doi: 10.1038/nrn755 . 11994752 10.1038/nrn755

[28] Corbetta M, Patel G, Shulman GL. The reorienting system of the human brain: from environment to theory of mind. Neuron. 2008;58(3):306–24. doi: 10.1016/j.neuron.2008.04.017 ; PubMed Central PMCID: PMCPMC2441869. 18466742 10.1016/j.neuron.2008.04.017 PMC2441869

[29] Fox MD, Raichle ME. Spontaneous fluctuations in brain activity observed with functional magnetic resonance imaging. Nat Rev Neurosci. 2007;8(9):700–11. doi: 10.1038/nrn2201 . 17704812 10.1038/nrn2201

[30] Weissman DH, Roberts KC, Visscher KM, Woldorff MG. The neural bases of momentary lapses in attention. Nat Neurosci. 2006;9(7):971–8. doi: 10.1038/nn1727 . 16767087 10.1038/nn1727

[31] Anderson BA, Folk CL, Courtney SM. Neural mechanisms of goal-contingent task disengagement: Response-irrelevant stimuli activate the default mode network. Cortex. 2016;81:221–30. doi: 10.1016/j.cortex.2016.05.006 ; PubMed Central PMCID: PMCPMC4958573. 27253724 10.1016/j.cortex.2016.05.006 PMC4958573

[32] Epshtein B, Lifshitz I, Ullman S. Image interpretation by a single bottom-up top-down cycle. Proc Natl Acad Sci U S A. 2008;105(38):14298–303. doi: 10.1073/pnas.0800968105 ; PubMed Central PMCID: PMCPMC2567169. 18796607 10.1073/pnas.0800968105 PMC2567169

[33] Roelfsema PR. Cortical algorithms for perceptual grouping. Annu Rev Neurosci. 2006;29:203–27. doi: 10.1146/annurev.neuro.29.051605.112939 . 16776584 10.1146/annurev.neuro.29.051605.112939

[34] Pooresmaeili A, Roelfsema PR. A growth-cone model for the spread of object-based attention during contour grouping. Curr Biol. 2014;24(24):2869–77. doi: 10.1016/j.cub.2014.10.007 . 25456446 10.1016/j.cub.2014.10.007

[35] Hochstein S, Ahissar M. View from the top: hierarchies and reverse hierarchies in the visual system. Neuron. 2002;36(5):791–804. . 12467584 10.1016/s0896-6273(02)01091-7

[36] Freedman DJ, Assad JA. Experience-dependent representation of visual categories in parietal cortex. Nature. 2006;443(7107):85–8. doi: 10.1038/nature05078 . 16936716 10.1038/nature05078

[37] Kveraga K, Boshyan J, Bar M. Magnocellular projections as the trigger of top-down facilitation in recognition. J Neurosci. 2007;27(48):13232–40. doi: 10.1523/JNEUROSCI.3481-07.2007 . 18045917 10.1523/JNEUROSCI.3481-07.2007 PMC6673387

[38] Kanai R, Bahrami B, Rees G. Human parietal cortex structure predicts individual differences in perceptual rivalry. Curr Biol. 2010;20(18):1626–30. doi: 10.1016/j.cub.2010.07.027 ; PubMed Central PMCID: PMCPMC2949566. 20727757 10.1016/j.cub.2010.07.027 PMC2949566

[39] Bisley JW, Goldberg ME. Attention, intention, and priority in the parietal lobe. Annu Rev Neurosci. 2010;33:1–21. doi: 10.1146/annurev-neuro-060909-152823 ; PubMed Central PMCID: PMCPMC3683564. 20192813 10.1146/annurev-neuro-060909-152823 PMC3683564

[40] Alvarez GA. Representing multiple objects as an ensemble enhances visual cognition. Trends Cogn Sci. 2011;15(3):122–31. doi: 10.1016/j.tics.2011.01.003 . 21292539 10.1016/j.tics.2011.01.003

[41] Vaziri-Pashkam M, Xu Y. Goal-Directed Visual Processing Differentially Impacts Human Ventral and Dorsal Visual Representations. J Neurosci. 2017;37(36):8767–82. doi: 10.1523/JNEUROSCI.3392-16.2017 ; PubMed Central PMCID: PMCPMC5588467. 28821655 10.1523/JNEUROSCI.3392-16.2017 PMC5588467

[42] Bisley JW, Krishna BS, Goldberg ME. A rapid and precise on-response in posterior parietal cortex. J Neurosci. 2004;24(8):1833–8. doi: 10.1523/JNEUROSCI.5007-03.2004 ; PubMed Central PMCID: PMCPMC2366207. 14985423 10.1523/JNEUROSCI.5007-03.2004 PMC2366207

[43] Mirpour K, Ong WS, Bisley JW. Microstimulation of posterior parietal cortex biases the selection of eye movement goals during search. J Neurophysiol. 2010;104(6):3021–8. doi: 10.1152/jn.00397.2010 ; PubMed Central PMCID: PMCPMC3007667. 20861428 10.1152/jn.00397.2010 PMC3007667

[44] Arcaro MJ, Pinsk MA, Kastner S. The Anatomical and Functional Organization of the Human Visual Pulvinar. J Neurosci. 2015;35(27):9848–71. doi: 10.1523/JNEUROSCI.1575-14.2015 ; PubMed Central PMCID: PMCPMC4495241. 26156987 10.1523/JNEUROSCI.1575-14.2015 PMC4495241

[45] Andersen RA, Asanuma C, Essick G, Siegel RM. Corticocortical connections of anatomically and physiologically defined subdivisions within the inferior parietal lobule. J Comp Neurol. 1990;296(1):65–113. doi: 10.1002/cne.902960106 . 2358530 10.1002/cne.902960106

[46] Ohla K, Busch NA, Herrmann CS. Early electrophysiological markers of visual awareness in the human brain. Neuroimage. 2007;37(4):1329–37. doi: 10.1016/j.neuroimage.2007.06.010 . 17656113 10.1016/j.neuroimage.2007.06.010

[47] Schoenfeld MA, Hopf JM, Merkel C, Heinze HJ, Hillyard SA. Object-based attention involves the sequential activation of feature-specific cortical modules. Nat Neurosci. 2014;17(4):619–24. doi: 10.1038/nn.3656 . 24561999 10.1038/nn.3656

[48] Driver J, Davis G, Russell C, Turatto M, Freeman E. Segmentation, attention and phenomenal visual objects. Cognition. 2001;80(1–2):61–95. . 11245840 10.1016/s0010-0277(00)00151-7

[49] Bogler C, Bode S, Haynes JD. Decoding successive computational stages of saliency processing. Curr Biol. 2011;21(19):1667–71. Epub 2011/10/04. doi: 10.1016/j.cub.2011.08.039 . 21962709 10.1016/j.cub.2011.08.039

[50] Mevorach C, Humphreys GW, Shalev L. Opposite biases in salience-based selection for the left and right posterior parietal cortex. Nat Neurosci. 2006;9(6):740–2. doi: 10.1038/nn1709 . 16699505 10.1038/nn1709

[51] Mevorach C, Shalev L, Allen HA, Humphreys GW. The left intraparietal sulcus modulates the selection of low salient stimuli. J Cogn Neurosci. 2009;21(2):303–15. doi: 10.1162/jocn.2009.21044 . 18564052 10.1162/jocn.2009.21044

[52] Smyrnis N, d'Avossa G, Theleritis C, Mantas A, Ozcan A, Evdokimidis I. Parallel processing of spatial and serial order information before moving to a remembered target. J Neurophysiol. 2005;93(6):3703–8. doi: 10.1152/jn.00972.2004 . 15625093 10.1152/jn.00972.2004

[53] Sestieri C, Pizzella V, Cianflone F, Luca Romani G, Corbetta M. Sequential activation of human oculomotor centers during planning of visually-guided eye movements: a combined fMRI-MEG study. Front Hum Neurosci. 2007;1:1 doi: 10.3389/neuro.09.001.2007 ; PubMed Central PMCID: PMCPMC2525985. 18958215 10.3389/neuro.09.001.2007 PMC2525985

[54] Bar M, Kassam KS, Ghuman AS, Boshyan J, Schmid AM, Dale AM, et al Top-down facilitation of visual recognition. Proc Natl Acad Sci U S A. 2006;103(2):449–54. doi: 10.1073/pnas.0507062103 ; PubMed Central PMCID: PMCPMC1326160. 16407167 10.1073/pnas.0507062103 PMC1326160

[55] Wagemans J, Feldman J, Gepshtein S, Kimchi R, Pomerantz JR, van der Helm PA, et al A century of Gestalt psychology in visual perception: II. Conceptual and theoretical foundations. Psychol Bull. 2012;138(6):1218–52. doi: 10.1037/a0029334 ; PubMed Central PMCID: PMCPMC3728284. 22845750 10.1037/a0029334 PMC3728284

[56] Chen L. Topological structure in visual perception. Science. 1982;218(4573):699–700. . 7134969 10.1126/science.7134969

[57] Kahneman D, Treisman A, Gibbs BJ. The reviewing of object files: object-specific integration of information. Cogn Psychol. 1992;24(2):175–219. . 1582172 10.1016/0010-0285(92)90007-o

[58] von der Heydt R. Figure-ground organization and the emergence of proto-objects in the visual cortex. Front Psychol. 2015;6:1695 doi: 10.3389/fpsyg.2015.01695 ; PubMed Central PMCID: PMCPMC4630502. 26579062 10.3389/fpsyg.2015.01695 PMC4630502

[59] Swettenham JB, Anderson SJ, Thai NJ. MEG responses to the perception of global structure within glass patterns. PLoS ONE. 2010;5(11):e13865 doi: 10.1371/journal.pone.0013865 ; PubMed Central PMCID: PMCPMC2974635. 21079764 10.1371/journal.pone.0013865 PMC2974635

[60] Altmann CF, Bulthoff HH, Kourtzi Z. Perceptual organization of local elements into global shapes in the human visual cortex. Curr Biol. 2003;13(4):342–9. . 12593802 10.1016/s0960-9822(03)00052-6

[61] Lalor EC, Foxe JJ. Visual evoked spread spectrum analysis (VESPA) responses to stimuli biased towards magnocellular and parvocellular pathways. Vision Res. 2009;49(1):127–33. doi: 10.1016/j.visres.2008.09.032 . 18977382 10.1016/j.visres.2008.09.032

[62] Van Dromme IC, Premereur E, Verhoef BE, Vanduffel W, Janssen P. Posterior Parietal Cortex Drives Inferotemporal Activations During Three-Dimensional Object Vision. PLoS Biol. 2016;14(4):e1002445 doi: 10.1371/journal.pbio.1002445 ; PubMed Central PMCID: PMCPMC4833303. 27082854 10.1371/journal.pbio.1002445 PMC4833303

