# Supplementary material for: Perceptual integration rapidly activates dorsal visual pathway to guide local processing in early visual areas
Source: PLoS Biol. 2017 Nov 30;15(11):e2003646. doi: 10.1371/journal.pbio.2003646 (PMC5726727; doi:10.1371/journal.pbio.2003646)
Supplement: S1 Text — (DOCX) [file pbio.2003646.s001.docx]

**Supplementary Materials and Methods**

**Participants**

Twenty subjects (11 females, aged 22–32 years) participated in experiment 1; 16 subjects (8 females, aged 19–29) participated in experiments 2; 16 subjects (8 females, aged 19–29) participated in experiments 3; 4 subjects participated the rate control experiment. Some subjects attended more than one experiment. All subjects had normal or corrected-to-normal vision and provided informed consent prior to the start of the experiment, which was approved by the Research Ethics Committee at Peking University (2015-03-05c2). All subjects were paid as compensation for their time.

**Stimuli and tasks**

The glass pattern stimuli were presented through a gamma-corrected projector/mirror system (60 Hz refreshing rate) at a fixed distance of 75 cm in front of the subject. The glass pattern stimuli consisted of 1,000 square dots (0.0125° × 0.0125°) that were randomly arranged on a mid-gray background (181 cd/m^2^) in a square aperture (20° × 20°) (Figure 1). Glass patterns with concentric or radial form structures were created by orienting the dot pairs (dot separation, 0.0625°) according to concentric or radial rules respectively. The proportion of rule-oriented dot pairs, defined as the global form coherence, could be systematically changed from 0 to 1 to modulate the saliency of the emerged global form property of the glass pattern stimuli accordingly. For example, it would be easy to perceive the global form of a glass pattern with 100% global form coherence, whereas a glass pattern with 1% global form coherence would be rather viewed as a unstructured noise pattern that does not contain any global form.

To extract the impulse brain response using TRF technique, the luminance and the global form coherence (i.e., reflecting the saliency of perceptual integration) were independently modulated in time throughout each trial. Specifically, in each 5 sec trial, the luminance (from black to white; 100 levels; mean luminance: 393.5 cd/m^2^) and the global form coherence (from 1% to 100%; 100 levels) of the glass pattern stimuli were respectively modulated at each refreshing frame, according to the corresponding temporal sequence that was randomly and independently generated for each trial (Figure 1, left panel). The stimulus temporal sequences were designed to have equal power at all frequencies. Each subject performed 120 trials, among which only 20 trials contained a target. Trials that contained no target were further analyzed, resulting in 100 trials for TRF calculation in each subject.

In Experiment 1, subjects were instructed to maintain fixation on the central point and detect whether the 5-s glass pattern contained a brief circular-to-radial global form change that appeared at a random time in the trial (i.e., global-form relevant task), by pressing the corresponding buttons (“Yes” or “No”) at the end of each trial.

In Experiment 2, subjects were instructed to maintain fixation on the central point and detect whether the 5-s glass pattern stimuli contained a brief overall large luminance change that appeared at a random time in the trial (i.e., global-form irrelevant task), by pressing the corresponding buttons (“Yes” or “No”) at the end of each trial.

Notably, in Experiment 1 and 2, to ensure that the subjects could clearly perceive and track the global form structure of the glass pattern visual stimuli, we slowed down the modulation rate of the global form coherence by temporally smoothing (8 points) the temporal sequence for global form coherence. In Experiment 3, subjects performed the same task as in Experiment 1, by detecting whether the 5-s glass pattern contained a brief circular-to-radial global form change that appeared at a random time in the trial. Meanwhile, different from Experiment 1, the stimulus temporal sequence for global form coherence was not temporally smoothed as done in Experiment 1 and 2. In the rate control experiment, the modulation rate of the luminance was slowed down, as done for global form coherence property, by temporally smoothing (8 points) the luminance temporal sequence.

**MEG data acquisition and preprocessing**

Neuromagnetic signals were recorded continuously with a 275 channel whole-heard MEG system (axial gradiometer SQUID-based sensory, CTF system, Canada) in a magnetically shielded room, using a sampling rate of 600 Hz and an online analog 50 Hz low-pass filter. Third-order synthetic gradiometer and linear drift corrections were applied to remove far-field noise. The relative localization of the head within the MEG helmet was recorded with three magnetic coils that were attached to the nasion and right and left preauricular points to coregister each individual head shape with the sensor coordinate system. Head position was acquired and checked at the beginning of each block to ensure that head movements did not exceed 0.2 cm. These landmarks enabled coregistration of the MEG activity with individual anatomical MRIs for further source localization analysis.

The MEG data were preprocessed with the FieldTrip toolbox in MATLAB (Donders Centre for Cognitive Neuroimaging, Nijmegen, The Netherlands). The data were bandpass filtered between 1 and 35 Hz offline. To verify the data quality and remove possible contamination from artifacts, such as cardiac, eye movements, blinks, and environmental noise, an independent component analysis (ICA) was performed. Next, the preprocessed MEG responses were downsampled to 60 Hz (same as the monitor refreshing rate) before TRF calculation. Furthermore, to avoid possible influence of the onset response, which may bias the estimated TRF results, we extracted the middle part of the 5-s MEG trial responses (0.5–4.5 s) for TRF calculation (Figure S1).

**Data analysis**

*TRF computation*

The mapping between the stimulus and the recorded MEG data were characterized by TRF response, which describes the brain’s response for unit transient in the stimulus temporal sequence. The TRF response is the forward linear model describing the relationship between the stimulus and neural response. The TRF describes the brain’s linear transformation of a stimulus input, S(t), to the neural response output, R(t), as R(t) = TRF*S(t), where * denotes the convolution operator. This forward linear model can reveal the timing and spatial information of the neural encoding process. The TRF functionally describes how the global form property and luminance property are transformed into cortical responses. Specifically, in each trial, the TRF responses for global form coherence (F-TRF) and luminance (L-TRF), as a function of temporal lag (-0.5 s ~ 0.4 s), were calculated from the same MEG recordings, based on the corresponding stimulus temporal sequences (global form coherence temporal sequence, luminance temporal sequence) for the trial, using normalized reversed correlations and a jackknife cross-validation procedure to minimize overfitting effects. The baseline activities (-0.5 s to 0 s) were calculated in order to obtain enough data for noise covariance matrix estimations in further source localization. Moreover, the TRF responses were baseline corrected by subtracting the averaged response during baseline period. This procedure was done for each of the 275 MEG channels in each subject. The calculated TRF response was then averaged over trials to represent the F-TRF and L-TRF responses in each MEG channel and in each subject. Note that the TRF represents an average measure of how the brain responds to a unit change in global form and luminance as a function of latency. The global form coherence and luminance values of the stimulus sequence and MEG signals were normalized before TRF calculation. The TRFs were in arbitrary units.

The temporal waveforms (i.e. Figure 2AB, Figure 4AB), which represent the root-mean-square (RMS) across all 275 MEG channels. A permutation test was then performed on the sensor-level RMS temporal waveforms. Specifically, we shuffled the relationship between trial response and the stimulus sequence and recalculated the L-TRF and F-TRF responses on the surrogate stimulus-data pairs for 1000 times so that we could get a 95% confidence interval (gray shades) for the RMS response at each time bin.

*TRF Source localization*

Each subject underwent anatomical MRI scans on a 3T Siemens Prisma scanner (voxel size: 1 mm^3^; flip angle: 9; echo time: 1.97 ms; repetition time: 2,300 ms; field of view: 256 × 256 × 176 mm) after MEG recordings. We used the digitizer data and three anatomical landmarks to align each subject’s MEG data with their individual anatomical MRI data. Next, to examine the source localization of TRF responses, for each subject, source modeling was performed with dSPM inverse modelling using MNE-Python tools to project the sensor-level TRF responses into the cortical surface, according to a realistic boundary element model. Finally, the source-level neuronal activation profile for each subject was normalized to MNI coordinates, which were then grand averaged across subjects.

To determine where and when the TRF responses showed statistically significant activations, we first performed two-tailed *t*-tests on the source-level TRF activations within consecutive 100-ms time windows. Next, a cluster-level permutation test across space and time was performed to correct multiple comparisons, resulting in statistically significant clusters in source space.

*ROI activation time courses and granger causality analysis*

Based on the source localization results in combination with anatomical landmarks, regions of interests (ROIs) for the F-TRF and L-TRF responses were separately defined and the corresponding activation time coursed were extracted. In Experiment 2, significantly activated ROIs belonging to DMN were grouped to represent DMN activity time course. Similarly, significantly activated ROIS belong to VAN were grouped to represent VAN activity time course.

Next, the activation time courses within each defined ROIs were extracted. To quantify the time points at which TRF showed significant activations, we conducted two-tailed *t*-tests for each time point between 0 and 300 ms for each ROI. Multiple comparisons were further corrected using Bonferroni correction.

Granger causality analysis was next performed on the extracted ROI-based activation time courses using the Multivariate Granger Causality toolbox. The theoretical and null distributions for all pairs were compared with Kolmogorov-Smirnov tests.
