# Supplementary figures and images for: Perceptual integration rapidly activates dorsal visual pathway to guide local processing in early visual areas
Source: PLoS Biol. 2017 Nov 30;15(11):e2003646. doi: 10.1371/journal.pbio.2003646 (PMC5726727; doi:10.1371/journal.pbio.2003646)

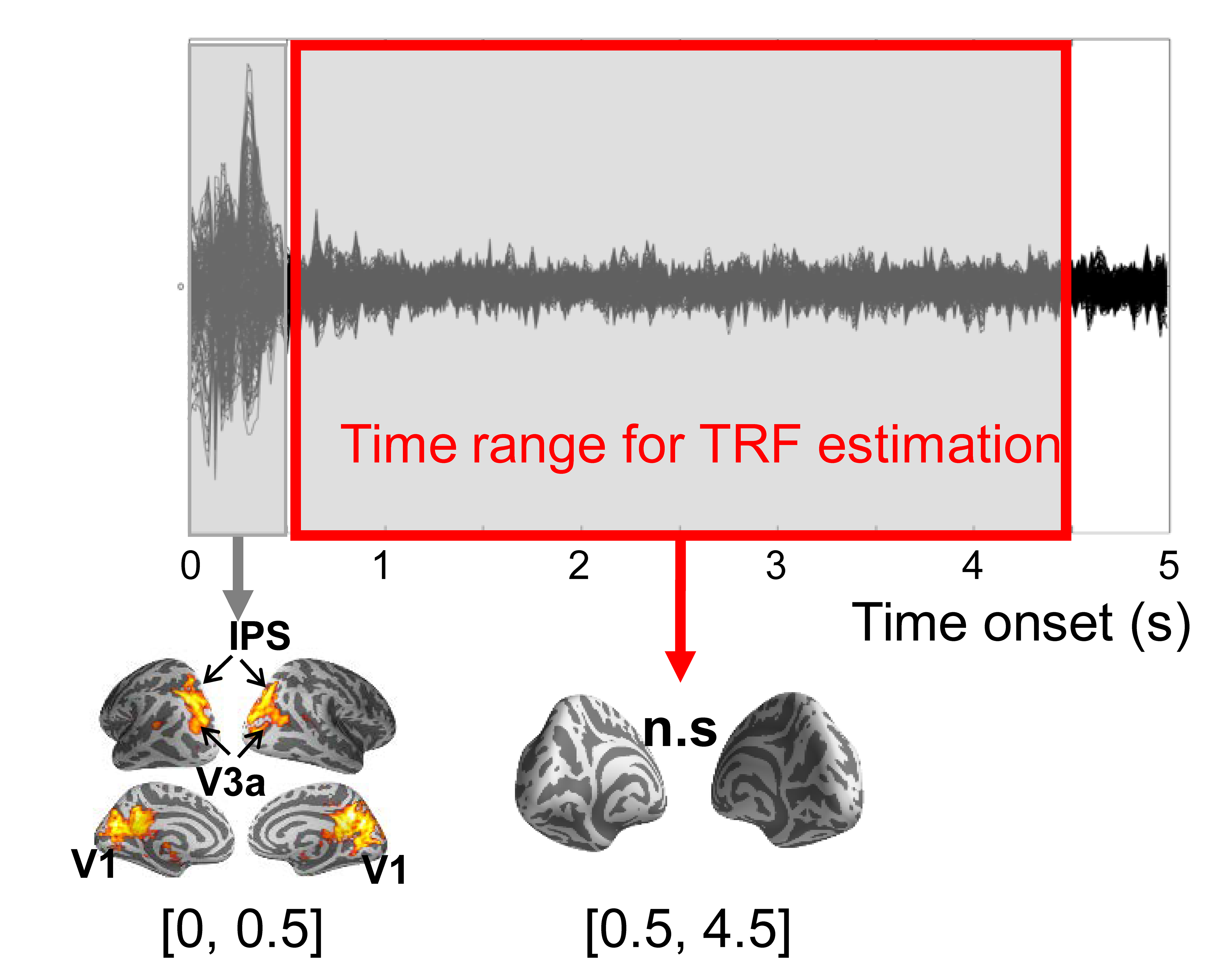

Supplement: S1 Fig — Top: ERFs for all MEG channels as a function of time (0–5 s) after trial onset. Bottom: Source localization of the initial ERF onset responses (0–0.5 s). To avoid possible influence from the onset and offset responses, which may bias the estimated TRF results, we extracted the middle part (red rectangle) of the 5-s MEG trial responses (0.5–4.5 s) for further TRF calculation. Notably, the data segments for further TRF calculation showed a rather noisy and flat response pattern. ERF, event-related magnetic field; MEG, magnetoencephalography; TRF, temporal response function. (TIF) [file pbio.2003646.s002.tif]

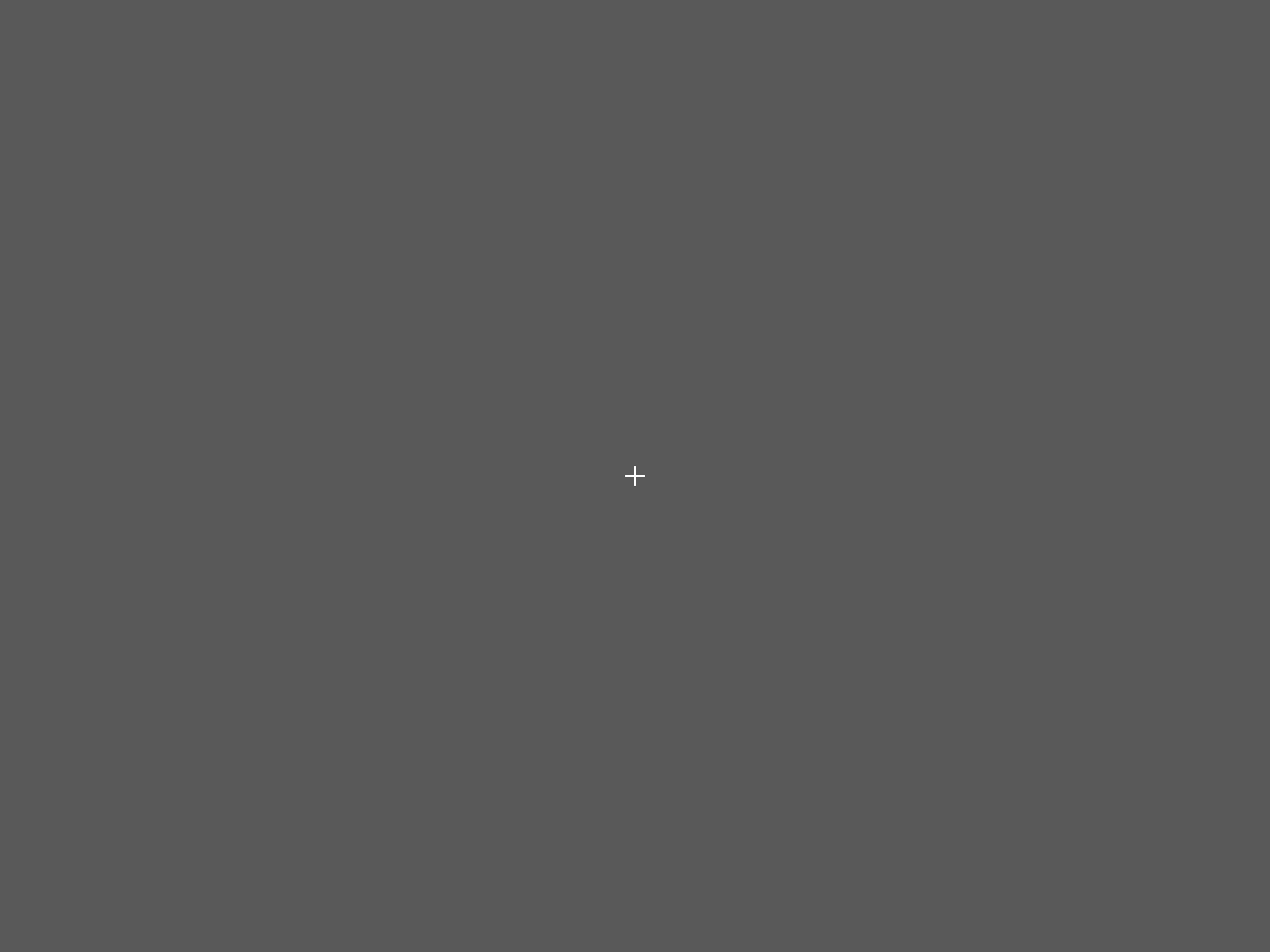

Supplement: S1 Movie — (GIF) [file pbio.2003646.s004.gif]
